# Direct Observation of the Developing Intra-Annual Density Fluctuation (IADF) for Scots Pine in Semiarid Siberian Belt Forest: External Stress Targets Cambium

**DOI:** 10.3390/plants15030348

**Published:** 2026-01-23

**Authors:** Yulia A. Kholdaenko, Natalia V. Karmanovskaya, Liliana V. Belokopytova, Dina F. Zhirnova, Nariman B. Mapitov, Eugene A. Vaganov, Elena A. Babushkina

**Affiliations:** 1 Institute of Ecology and Geography, Siberian Federal University, 79 Svobodny Prospect, Krasnoyarsk 660041, Russia; kropacheva_yulechka@mail.ru (Y.A.K.); dina-zhirnova@mail.ru (D.F.Z.); eavaganov@hotmail.com (E.A.V.); babushkina70@mail.ru (E.A.B.); 2 Khakass Technical Institute, Siberian Federal University, 27 Schetinkina Street, Abakan 655017, Russia; 3 Ecological Educational Center “Noosphere”, Fedorovsky Polar State University, 7 50-let-Oktyabrya Street, Norilsk 663310, Russia; karmanovskayanv@norvuz.ru; 4 Department of Biology and Ecology, Toraighyrov University, 64 Lomov Street, Pavlodar 140008, Kazakhstan; mapitov@mail.ru; 5 Department of Dendroecology, V.N. Sukachev Institute of Forest, Siberian Branch of the Russian Academy of Science, 50/28 Akademgorodok, Krasnoyarsk 660036, Russia

**Keywords:** phenology of xylogenesis, tree-ring formation, false ring, tracheidograms, mechanistic VS-model of tree growth, *Pinus sylvestris* L., water stress, Southern Siberia

## Abstract

Long-term observations of the seasonal growth of Scots pine (*Pinus sylvestris* L.) tree rings in the arid conditions of the Khakass-Minusinsk Basin (southern Siberia) revealed that in 2024, trees had formed a tree ring with a typical intra-annual density fluctuation (IADF) in the transition wood. An analysis of the timing and causes of this wood structure anomaly was conducted using a combination of three approaches: (1) analyzing images of cross-sections of the forming tree ring throughout the season; (2) comparing the timing of anomalous cells’ differentiation with daily climate data; (3) comparing seasonal growth observations with calculated characteristics of the modeled growth rate and its derivatives: soil moisture and transpiration. We found that during the most severe heat wave and drought (from 22 June to 9 July), the last normal earlywood cells were yet expanding, IADF cells were being produced in the cambial zone, and the first of them began expansion, while normal cells began being produced again immediately after the subsiding of environmental stress. Apparently, low soil moisture and very high temperatures mainly impacted cells in the cambial zone, marking it as the primary target of external factors influencing tree-ring formation and structure, which is important for dendroclimatology and digital wood anatomy. This result is supported by both indirect and limited direct evidence from other sources.

## 1. Introduction

Measurements of intra-annual density fluctuations (IADFs) in wood—their frequency and relative locations within annual rings—are usually aimed at identifying the factors and events that cause the formation of abnormal “false” rings in woody plants growing under different conditions [1,2,3,4,5,6]. Numerous publications show that the reasons for the formation of false rings can be different: intra-seasonal drought, a sharp drop in temperature (for example, recurrent frosts), and defoliation by phytophages [4,7,8,9,10,11]. The most common opinion is that the false ring is formed under the influence of turgor changes in the cell expansion zone [11,12,13,14]. This opinion is based on a simple assumption: cells in the forming annual ring perceive an external signal while they are in the corresponding differentiation zone [15]. However, there is an alternative working hypothesis that the primary target of external influence is the cambial zone [8,15,16,17,18]. The simplest way to prove if cell structure is determined by conditions during the production or differentiation of those cells is to perform direct phenological observation of xylogenesis (process of wood growth and development) over the season with distinctive periods of stress leading to the formation of well-expressed multi-cellular IADF.

The question we raised in this study is of fundamental importance in dendroclimatology for interpreting the relationships between tree radial growth and key climatic factors. If each phase of xylem differentiation depends on current seasonal conditions, then solving both the direct and inverse problems of dendroclimatic analysis becomes extremely complex, if not impossible. However, what if there is a key phase in the seasonal cycle of tree-ring formation that triggers and transmits the influence of external conditions into subsequent differentiation? In this case, a real opportunity arises not only to focus the external influences on this key phase, but also to construct an experimental scheme for sequentially analyzing the initiation of a genetic program, ultimately leading to the formation of a specific xylem anatomy as a vascular system for many years of the tree’s life.

Since 2013, the Laboratory of Dendroecology and Ecological Monitoring (Khakass Technical Institute, Siberian Federal University) has been conducting long-term observations of the intra-seasonal growth and development of tree-ring tracheids in conifers of southern Siberia, including Scots pine (*Pinus sylvestris* L.), under moisture-deficient conditions [19]. This drought-tolerant species is a forest-forming tree in the southern and lower reaches of the Siberian boreal forests and is widely distributed across Eurasia. Therefore, it is an excellent candidate for studying the effects of water stress, including droughts. In this study, using the example of IADF formation observed in Scots pine in 2024, we determined which zone of the developing tree ring perceives drought signals. To do this, we (1) analyzed the kinetics of differentiation (estimated intervals of particular developmental stages) for cells that make up IADF, as well as for other tree-ring zones, (2) identified intra-seasonal temporal limits of climatic stress based on climate data and the Vaganov-Shashkin model (VS-model), and (3) compared the timeframes of tracheid formation for cells before, within, and after IADF, with conditions favorable or limiting pine growth.

## 2. Results

### 2.1. IADF Development in the Fast-Growing Pine Tree

Among seven sampled trees, for tree rings formed in 2024, variation in the tracheid number per radial file (and tree-ring width) was quite large despite similar tree sizes: N = 18–52 cells. According to this variation, we considered trees with the largest cell number as fast-growing, while trees with smaller cell numbers (18–19 cells) were slow-growing in this particular year.

**Figure 1:**
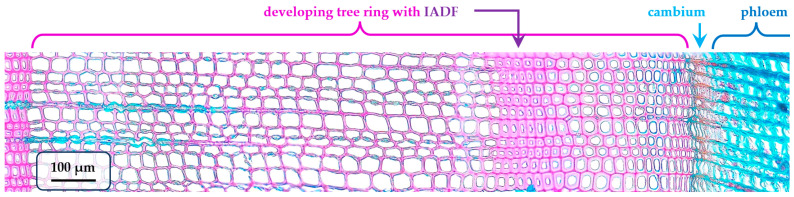
Intra-annual density fluctuation (IADF) in the developing tree ring of Scots pine (*Pinus sylvestris* L.) on 10 September 2024, for fast-growing tree #5. On the microphotograph of wood cross-section, lignified wood matter is stained maroon and non-lignified cells are stained blue. IADF (band of small thick-walled tracheids) is formed in the end of earlywood and followed by larger tracheids of transition wood. The last several cells of tree ring are not fully lignified on this date, but cell division and growth by expansion already have ceased. Developing ring of the current year, small cells of inactive cambium, and phloem are marked and labeled.

The abnormal structure of wood is very clearly expressed in the ring of the fast-growing pine tree, if we consider the moment of almost complete completion of seasonal growth in the analyzed year, on 10 September (Figure 1).

**Figure 2:**
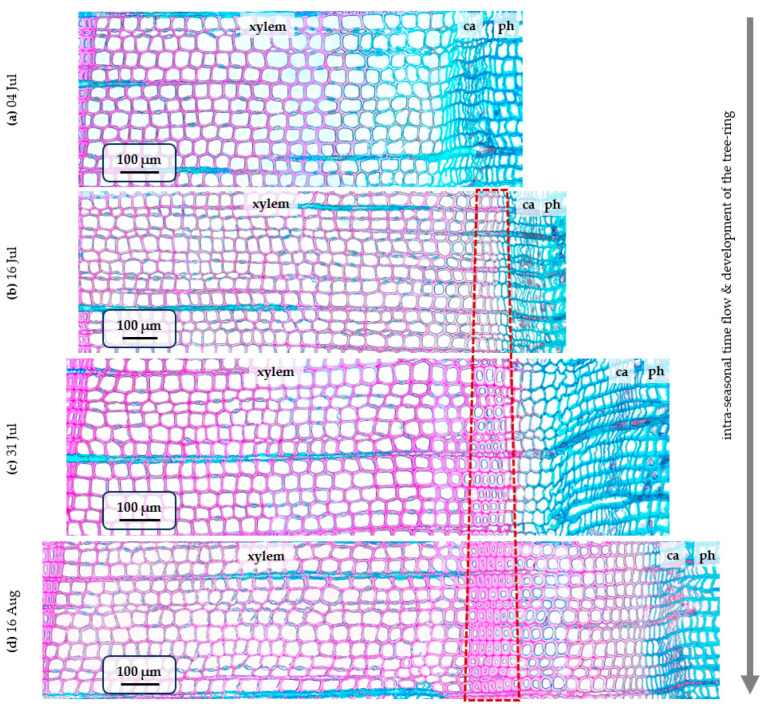
Consequential observations of the developing tree ring of Scots pine with IADF from Figure 1: microphotographs of the stained cross-sections made from samples from tree #5 collected on 4 July (**a**), 16 July (**b**), 31 July (**c**), and 16 August (**d**). Vertical gray arrow marks temporal and developmental axis. Cross-sections are colored (stained) the same as on Figure 1. Location of the anomalous tracheids (IADF) is approximately marked with dashed trapezoid; developing xylem, cambial zone (ca), and phloem cells (ph) are labeled. Note that number of the produced tracheids per radial file can vary not only with time (i.e., new cells being produced during season), but also between sampled radii (i.e., eccentricity of growth).

The structural pattern of this ring is the formation of anomalous tracheids, i.e., IADF, after 24–25 large thin-walled cells of typical earlywood [8]. The identified anomaly in the radial direction consists of 7–8 tracheids similar to those in the end of latewood—with very much thickened walls and reduced lumens compared to previous cells—and also has an indistinctive border with subsequent cells of transitional wood. After the false ring, larger but relatively thick-walled cells (slightly smaller than in earlywood) formed, indicating the onset of better growth conditions. Moreover, the number of cambial cells after the cessation of their activity stays at five to six, as noted in our previous study [20]. A more detailed picture of the tree-ring formation in 2024 is shown by a sequence of wood cross-sections from samples obtained at certain moments within the growth season (Figure 2).

It is easy to see that by 4 July, the cells forming the IADF are not differentiating yet, considering that the number of developing xylem cells at this moment (22–24) is a little less than the number of normal earlywood tracheids in the later photographs. However, by 16 July, there are already 2–3 layers of partially lignified cells that can be classified as part of the IADF. Adjacent to these on the cambium side are two more layers of cells growing by expansion, non-lignified but larger than the cambium cells. By 31 July, the number of small mature and maturing tracheids that can be classified as IADF reaches 6–7. In the same cross-section, larger cells (non-lignified) are present in the expansion zone and 1–2 layers of such cells in the maturation zone (with partially lignified but still thin cell walls), which form the transition zone. By 16 August, the false ring had fully developed, and after it, cambium produced another 14–15 cells, the first of which resembled earlywood cells in radial diameter, but then traditionally completed the ring structure with small thick-walled cells typical of latewood. In total, in 2024, the annual ring of this tree contained an average number of tracheids per radial file (N) of 52.

So, let us summarize the stages of IADF formation at different calendar dates. By mid-August, the false ring in the pine wood consists entirely of mature tracheids and is anatomically indistinguishable from that in a cross-section obtained in September (Figure 1). By late July, not all cells in the IADF have yet completed the lignification process. By mid-July, IADF formation exhibits a pattern indistinguishable from the normal transition to latewood: the first two to three layers of IADF cells have reduced radial diameters but are already being lignified, and the tracheids adjacent to them on the cambium side are comparable in size. Finally, at the beginning of July, the cells that will form the false ring are still in the cambial zone.

### 2.2. IADF Features in Tree Rings of Different Widths

**Figure 3:**
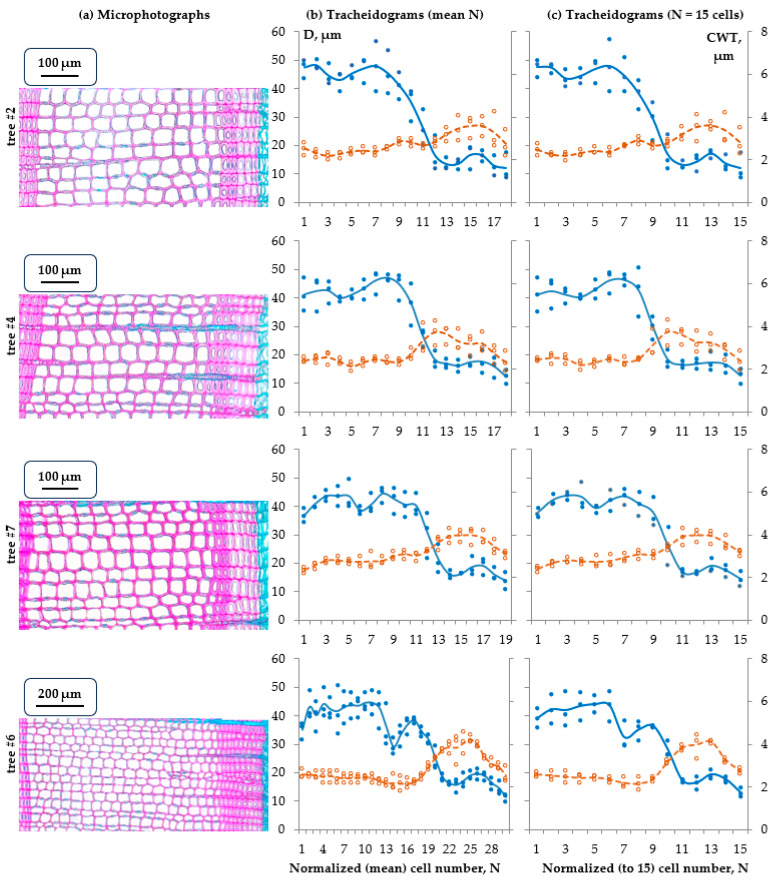

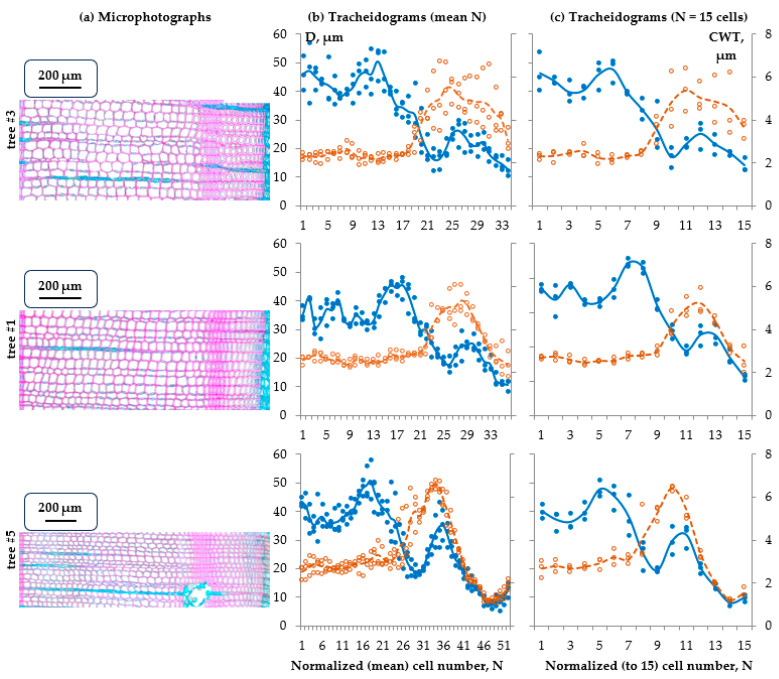
Fully developed tree rings of seven Scots pine trees on 10 September, 2024, arranged in order of increasing N: stained microphotographs (**a**); tracheidograms, i.e., measurements of cell radial diameter (D, solid line and filled dots, blue) and cell wall thickness (CWT, dotted line and empty dots, orange) along three radial files of tracheids and their averages, normalized for mean cell number per radial file (N) for this ring (**b**) and for N = 15 cells (**c**). Cross-sections are colored (stained) the same as in Figure 1. On tracheidograms, lines represent average dynamics while dots represent measurements for individual radial files of tracheids.

In Figure 3, we compared the tree-ring structure for seven studied pine trees on 10 September, when their seasonal growth, including cell division and differentiation, was close to complete. Stained cross-sections are shown on the left, with the corresponding tracheidograms normalized to the average number of cells in the three measured radial files in the center. Tracheidograms of these same rings, normalized to the common N = 15 cells, are shown on the right [8,21]. It is evident that despite the diversity of cell production in rings formed in different trees in 2024, from 18 to 52 per radial file, all measured tracheidograms show the presence of IADF to varying degrees (Table 1). The arrangement of cross-sections and tracheidograms in order of increasing cell production demonstrates a strengthening in the severity and detail of the anomaly.

As cell production over the course of a season increases, the number of cells in each of the identified zones (before, during, and after IADF formation) increases approximately proportionally. The more cells that are in a ring, the more detailed is the observed tracheidogram’s description of its structure. For example, a slight decrease in the radial cell sizes in the middle of the earlywood zone is most clearly evident in the tracheidograms of the fast-growing trees, containing 34, 36, and 52 cells. In terms of radial cell sizes, during the period of somewhat improved growth conditions after the false ring, changes are least noticeable in slow-growing trees forming 18–19 cells during the studied season.

Nevertheless, the normalized tracheidograms show meaningful similarities in the structure of tree rings across different trees in the study area, particularly in the relative number (proportion) of typically earlywood cells formed before the IADF and cells formed after it. The average tracheidograms demonstrate that on average 65% of cells in the ring have formed before the IADF. The anomaly itself contains 6–12% of cells, or 9% on average, and the latewood afterward contains about 25% of tracheids. Of all these proportions, only the zone after IADF increases with N significantly at *p* < 0.05 (*r* = 0.86), while the decrease in earlywood is not significant (*r* = −0.72) and the share of IADF is quite stable (*r* = −0.16). Interestingly, the maximum cell wall thickness in formed rings is positively correlated with cell production (correlation coefficient *r* = 0.97, significance level *p* < 0.0005) and increases drastically when comparing annual rings with N = 18–19 cells (3.5–4.0 µm) and N = 34–52 cells (5.3–6.3 µm). Normalizing tracheidograms to the same N, despite losing some details, shows a commonality of these patterns too.

### 2.3. Environmental Intra-Seasonal Fluctuations

**Figure 4:**
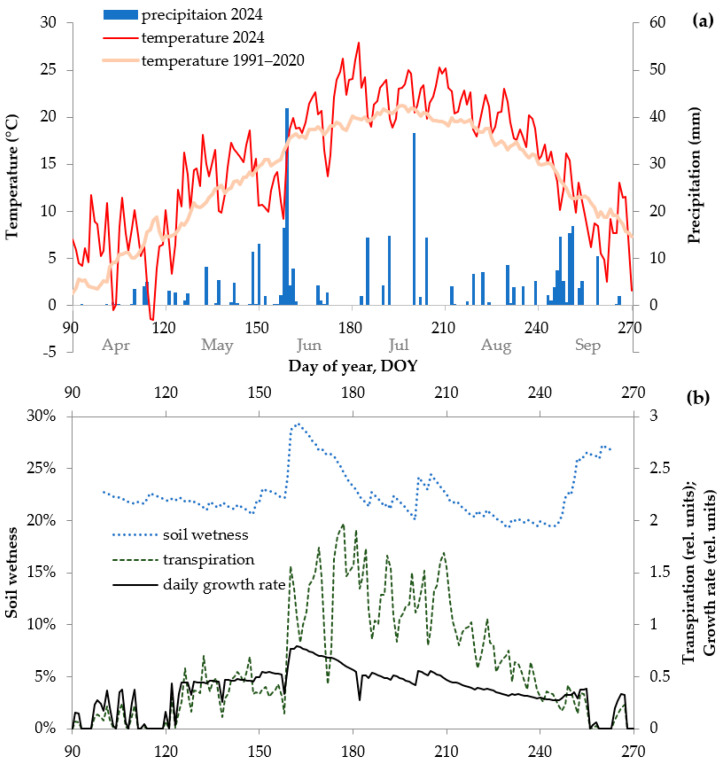
Dynamics of environmental factors over vegetative season (90th–270th day of year, DOY; i.e., ca. April–September) in 2024 in the study area: (**a**) daily amount of precipitation (blue bars) and mean temperature (thin red line) at the Minusinsk climatic station; for comparison, climatic norm of mean temperature averaged for 1991–2020 is presented (thick pink line); (**b**) soil wetness (dotted blue line), transpiration of pine trees (dashed green line), and their relative daily growth rate (solid black line) simulated in Vaganov-Shashkin model (VS-model) from daily climatic data.

In the absence of disturbances in the study area, the main external factors influencing the seasonal growth kinetics are the climatic variables; therefore, we investigated the daily dynamics of air temperature and precipitation (Figure 4a). According to direct observations, the transition of the first tracheids to the expansion zone in 2024 began between 8 May (only cambial cells present) and 21 May (several expanding tracheids). Apparently, the onset of cambial activity (swelling and subsequent division of cambial initials), which occurred at some time between 26 April (dormant cambium) and 8 May (increased cell number in cambial zone), roughly corresponded to the daily mean temperature rise since 3 May above 10 and up to 18 °C. However, in the first decade of June, a sharp drop in temperature was observed: 11 °C on average from 29 May to 6 June, while the climatic norms over the previous 30-year period of 1991–2020 for this intra-seasonal interval were about 15 °C. In the second decade of June, increased soil moisture after summarily 73 mm of rainfall over 5–9 June was accompanied by an increase in temperature a little above the climatic norm since 8 June, which provided favorable (warm and wet) growth conditions for the arid biomes of Khakassia. On the other hand, the subsequent period of the season (late June and early July) was characterized by a sharp and the most significant increase in temperature (22–29 °C for most days from 22 June to 9 July, whereas norm values were 19–20 °C) and a quite low amount of precipitation during and before this heat wave (less than 30 mm over the monthly interval from 10 June to 9 July, which is less than half of the norm value of 70 mm). That means the occurrence of severe drought. Since 10 July, the heat wave partially subsided and 15 mm of rain fell. Then, heavy rainfalls of 37 mm on 18 July and 14 mm on 22 July partially alleviated the moisture deficit and allowed the growing season to be completed without further severe stress despite the temperatures in August still being about 2 °C above the norm.

A comparison of the seasonal climate diagram with the tracheidogram and seasonal growth kinetics of the widest ring (52 cells) revealed that a slight decrease in radial cell sizes in the middle of the earlywood coincided with a significant drop in temperature in early June. The local minimum of cell size occurs about the 10th cell of the raw tracheidogram (Figure 3b), but on 4 June the direct observation registered on average 12–13 cells with radial diameters 16–17 μm and larger. This means that cells transitioning to the expansion stage during the cold spell in 29 May–6 June ended being smaller than those before or after them.

During the subsequent period of the season, the precipitation and temperature were quite optimal for growth, and several layers of earlywood cells with large radial diameters formed. However, the later heat wave and drought from 22 June to 9 July have to be compared with a sharp slowdown in the production and growth of new cells and the formation of IADF. Apparently, in the middle of the most severe drought, on 4 July, the first IADF cells were being produced, while a week after the subsiding of the heat wave, on 16 July, the first IADF cells were beginning secondary cell wall deposition while most of them were yet expanding. The production of IADF cells ended between 4 and 16 July, closer to the latter date. The end of cell expansion for the last IADF cells occurred between 16 and 31 July; they finished cell wall deposition and lignification during the first half of August, long after the end of the most severe stress. Overall, the climatic dynamics and seasonal growth kinetics over July and August for cells belonging to earlywood, IADF, and normal transition and latewood zones together are summarized in Table 2.

Larger transitional and then normal-sized latewood cells formed after IADF cells, according to direct observations, were being produced in the cambial zone from about 16 July to the second half of August, since on 28 August the last cells were at the expansion stage.

**Figure A1:**
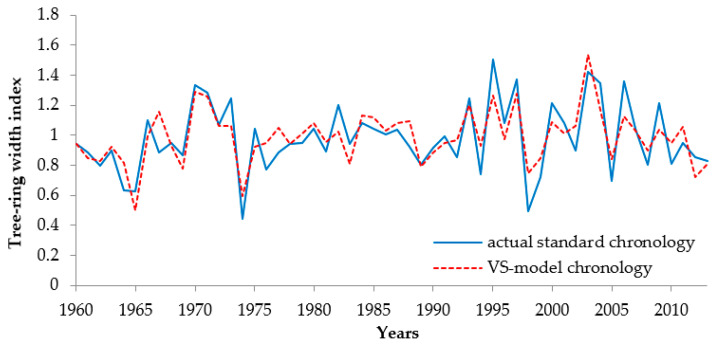
Long-term dynamics of Scots pine tree-ring width: actual local standard chronology (blue solid line) and VS-model chronology (red dashed line).

To support these comparisons, we used calculations in a process-based VS-model, which allows us to quantitatively describe the seasonal kinetics of radial tree-ring growth [8,22,23,24,25,26,27]. The actual local tree-ring width chronology fits well with the model one (Figure A1). The VS-model was parameterized over the period from 1960 to 2013, for which the following statistical estimates were obtained: the correlation coefficient between the actual and model chronologies *r* = 0.84, the synchronicity coefficient GLK = 83%, and the root mean square error RMSE = 0.126. Such statistical estimates of convergence with the actual growth indicate the high quality of the model. There are still some uncertainties that can lead to deviations of the modeled growth rate curves and environmental variables from the actual ones. However, the daily resolution of the input data means that those uncertainties are in regard to the intensity of variation in the calculated curves, but its timing is set—which is precisely what we are interested in here.

Using the fitted parameters’ values (Table A1), the VS-model calculated the intra-seasonal daily tree growth rate kinetics for each year, as well as the parameters of interest for this study—soil moisture dynamics and relative tree transpiration (Figure 4b). These parameters may be closely related to the formation of the false ring.

Daily model calculations for 2024 showed that pine growth commenced when, based on the fitted parameters, the temperature consistently reached its lower threshold for growth of 7 °C (i.e., the sum of temperatures over 10 days was 70 °C). The growth rate (cell production) in the first decade of June was lower than its increase which should occur in the lengthening days of that period, due to low temperatures, but it then promptly increased to the maximum in the second half of June. This period corresponded to the formation of typical earlywood tracheids.

Daily curves of the model-derived variables demonstrate that the heat wave in the third decade of June and early July, under conditions of low precipitation, increased moisture loss through tree transpiration and evaporation, leading to a sharp decrease in soil moisture (Figure 4b). The combination of these factors can be considered a severe intra-seasonal water stress, which was received as an external signal by cells of the developing tree ring even more so than the growth rate in general.

## 3. Discussion

Most studies of IADF have been conducted for semiarid tree growth conditions, particularly in the Mediterranean climate exhibiting clear moisture gradients. Indirect estimates of the timing of IADF formation are common in studies assessing its frequency and occurrence conditions. For example, for *Pinus pinea* growing in southern Portugal, an interesting study was conducted on IADF classification, indirectly accounting for both the severity of water stress (comparing coastal and inland sites) and the relative timing of IADF formation within the season [28]. It was shown that the highest frequency of these anomalies is observed in latewood, and the IADF types by location within the ring adopted by the authors correlated with stress seasonality based on their location. For example, E+ (end of earlywood) was associated with early summer precipitation events, but types L (latewood) and L+ (end of latewood) were associated with autumn precipitation. For *Pinus pinaster* growing in Spain, it was found that the frequency of IADF was higher in young trees, but radial growth was negatively correlated with its presence [1]. Similar studies in northwestern Spain revealed that the frequency of IADF decreased with increasing site elevation [3]. In coastal stands, IADF formation was stimulated by cool May–August, while in inland areas, dry May–July caused IADF. A study of the causes of false ring formation in *Pinus banksiana* tree rings in Michigan revealed its significant relationship with the canopy: codominant and intermediate trees had more false rings than suppressed trees [29].

Thus, among other patterns, direct and indirect evidence was obtained of a connection between the frequency and severity of IADF and the rate of tree growth: in fast-growing trees (young, dominant, growing in warmer conditions), obvious disturbances in the wood structure were detected more often [30,31,32]. This is important since in the multi-year project of observing the phenology of xylogenesis in the study area, the strategy of tree selection was concentrated on middle-aged, dominant, and thus relatively fast-growing trees to provide more detailed intra-seasonal records. Such a strategy lessens the variability in phenological dates between trees by homogenizing tree size and social standing, but necessitates a separate investigation of those factors’ potential input in the climatic sensitivity of various zones in the developing ring. The small sample size and limited variability of the growth rate (absence of cell numbers below 18) highlight the necessity of further research, too.

There is no doubt that most studies analyzing the frequency and causes of IADF formation are related to intra-seasonal droughts. These results are supported, in particular, by studies measuring the frequency of false rings in jack pine (*Pinus banksiana*) and black spruce (*Picea mariana*) trees in Manitoba, Canada, or yellow pine (*Pinus ponderosa*) in the southwestern USA, where severe summer droughts produced a higher frequency of false rings [33,34].

When investigating the impact of environmental factors on wood formation, there is an issue that the external signal perceived by cells during certain stages of differentiation can be recorded by the resulting characteristics of the same stage and/or of subsequent stage(s), meaning a delay or transfer of impact to the next stage. This means that in principle, there could be various combinations of sensitive stage(s) when the stress signal is perceived and the same and/or further stage(s) when it is recorded.

For the obvious anatomical anomaly with known environmental causes, like drought-induced IADF, a comparison of the cell differentiation phenology for cells comprising IADF with a timeframe of environmental stress allows us to answer the questions of which stages are sensitive to stress and which ones record them in wood anatomy. In the current study, we determined that, at least for Scots pine in a continental semiarid climate, the stress signal from heat and drought was perceived by the cells being produced. On the contrary, cells being in the latter stages of differentiation during stressful intervals proved to not be sensitive to water or heat stress and ended as normal earlywood, possibly by compensating the slower rate of development with its longer duration [35]. The smaller size and thicker cell walls of the IADF tracheids indicate that the stress signal was transferred to both further stages of cell differentiation and recorded in their results. Also, the formation of non-anomalous cells typical for transition wood and latewood, which began as soon as the most severe stress subsided, indicated that the formation of small thick-walled cells of IADF was a reaction not to the mild stress occurring in 2024 from mid-July to late August but exactly on the short-term severe heat and drought combination.

Logically, the result of cambial activity is the cell number per radial file formed during the whole season (tree ring) or its part (zone of the ring, IADF, among others). In addition to anatomical measurements, the cell division rate seasonal curve can be calculated during further research—and, if it had also slowed during a severe stress period, as it was reported to occur in some other studies [34,35,36,37], it would mean also recording the stress signal by those cells immediately, as a result of this same stage of xylogenesis.

Unfortunately, direct observations of the phenology of false ring formation during the course of a season are limited. Therefore, until now, there has been no clear answer to the question of which phase of tracheid differentiation (cambial zone or growth by expansion) is the primary recipient of the drought signal. Most studies rely on post hoc measurements of tracheid sizes in tree rings formed in years with intra-seasonal drought [38,39,40]. For example, tracheidograms of *Juniperus thurifera* tree rings showed a clear reduction in tracheid radial sizes in dry habitats [39]. For the results of seasonal growth observations for ponderosa pine (*Pinus ponderosa*) and Douglas fir (*Pseudotsuga menziesii*) in a mixed conifer forest, the authors asked several questions: what were the cell-by-cell timing and durations in the phases of wood development in 2014 and how did these seasonal patterns relate to environmental conditions during the growing season [34]? For ponderosa pine trees forming a false ring, the first impact of drought was seen in the cell expansion phase and only then in cambial activity, albeit it was concluded from the numbers of cells in respective phases during and after drought. However, recovery from drought was associated with recovery first in cambial activity. Bimodal cambial activity was also associated with moisture availability. Those results are somewhat contradictory and do not agree with those obtained in the current study, where during the most severe drought interval we observed the expansion of the last normal earlywood cells and the production and beginning of expansion for IADF cells, while all cells produced since the lessening of stress ended as normal (of typical size for respective tree-ring zone). The conclusions of Morino et al. [34] also diverge from earlier comparisons of seasonal data on pine tree-ring formation and its modeling under semiarid conditions [38]. However, that work also recorded the first normal cell after the IADF beginning expansion stage within 1–2 weeks after the conclusion of drought, which is fully consistent with our data. This ambiguity indicates the need for further research and a search for the truth in this area.

We believe that one of the possible reasons for ambiguity is the type of climate, because the Mediterranean semiarid climate observed by Morino et al. [34] (dry summer and mild winter frosts) yields clear bimodal cambial activity patterns in local conifer species, quite different from the normally unimodal growth observed here in the continental climate (maximum precipitation in summer and harsh winter). It can also be compounded by species-specific adaptation to the Mediterranean climate, since local species of conifers are more prone to bimodal xylogenesis than Scots pine [41].

One of the older studies on the formation of false rings in conifers clearly demonstrated that the appearance of smaller cells lags behind the minimum soil moisture by almost two weeks [42]. It is noteworthy that two weeks is the interval required for xylem mother cells to move from the cambium zone to the cell expansion zone [43], so this evidence also supports our observations. Also, it is interesting that a detailed examination of 313 trees of black pine (*Pinus nigra*) from 29 sites from the Viennese basin revealed that false rings are significantly related to May precipitation, and a high frequency of false rings was found under conditions of wet April, dry May, and wet June [7].

Overall, the problem of false ring formation is of interest not only to dendroclimatologists but also to researchers attempting to develop a theoretical basis for seasonal tree ring formation. One interesting study in this area combines technical innovations in measuring the anatomical characteristics of tree rings with the development of a model for the dynamics of tree water regime and carbohydrate supply to growing tissues [44]. Also of interest are experiments inducing a soil moisture deficit and then measuring tree ring formation in *Cryptomeria japonica* trees under increasing water stress [45].

Finally, as indirect evidence that the cambial zone is the acceptor of external signals (especially water stress), we present data from the monograph by Vaganov et al. [8] (p. 124). In Figure 4.10 of that book, a tracheidogram of a *Pinus rigida* tree ring is shown, demonstrating the rare occurrence of two false rings in one season. The main interesting detail of the tracheidogram is the fast transition from small cells back to typical large earlywood tracheids that coincided with the onset of the rainy season. Later authors of the book focused on an evident detail: if the effect (water stress) was felt in expanding cells, the transition in size would have been gradual, but it was not. Indirectly, those previous data support our hypothesis about the cambial zone as the main target of external influences on growth and tree-ring formation [16].

So, we are presenting here one of the first direct observations of conifer tracheids perceiving severe climatic stress while being produced in cambium rather than in later stages of their differentiation. Of course, it opens many other questions. In particular, there is the question of whether any factors creating variation in the phenology of xylogenesis are interacting in any way with this observed pattern, especially considering the presence of controversial or ambiguous data that we mentioned here.

There are many avenues to investigate, like tree size, age, and social standing. We believe that this observed pattern can be shared across various conifer species and climates, based on the supporting evidence. Also, as for the various environmental stresses, the comparative sensibility of the differentiation stages to them should be investigated considering their nature, severity, and seasonality.

## 4. Materials and Methods

Observations of the seasonal growth of Scots pine are being conducted in the semiarid Khakass-Minusinsk Basin in southern Siberia beginning from 2013. The regional climate is sharply continental; according to data from the nearest weather station in Minusinsk (53°41′ N 91°40′ E, 250 m a.s.l., 1936–2021, https://meteo.ru; accessed on 28 November 2025), the average annual temperature is +1.3 °C and annual precipitation is 350 mm.

Sampling was carried out in isolated pine forest (belt forest) in the steppe zone, near the city of Minusinsk (53°42′ N 91°38′ E, 270 m a.s.l.). The selected plot of natural forest consists purely of *Pinus sylvestris* trees of varying ages from saplings to mature (up to ca. 100–130 years old). This is relatively sparse standing (distances between neighboring trees are mainly 3 to 10 m, but canopies cover 80–90% of area), growing on sandy eolian soils, with a predominantly flat topography (no significant slopes). For direct measurements of the seasonal growth kinetics, seven mature (middle-aged as estimated by size and crown structure), undamaged, dominant pine trees without close neighbors (within 2 m distance) were selected in the stand inferior. Variation in the trunk size was minimized; DBH (diameter at breast height) of selected trees was about 40 to 50 cm (about largest for the sampling site). Small sampling size of seven trees is typical for direct observations of seasonal kinetics, as well as for wood anatomical studies in general, due to extremely time- and resource-consuming sample processing procedure.

Samples (mini-cores containing 2–3 growth rings) were collected every 14–15 days from late April to late September. For this study, samples of 2024 were used. A sampling tool similar to Trephor [46] but with a larger diameter of 4 mm was used. Sampling was performed throughout the season from the sunny side of the trunk, successively shifting the sampling location diagonally by ~5 cm. The choice of sampling direction was determined by considerations of trunk eccentricity (faster tree growth from the illuminated side on flat terrain), meaning a more detailed record of the seasonal kinetics. Thin (12–15 μm) transverse wood sections were obtained from the samples for measurement using a Microm HM340E rotary microtome (Thermo Fisher Scientific, Waltham, MA, USA). Cross-sections were stained with two contrast dyes (astra blue and safranin) to separate tracheids in the cell expansion and maturation phases: the red safranin binds only to lignin-containing cell walls, while astra blue stains all tissues uniformly. Microphotographs at 400× magnification were taken using a biological microscope BX43 (Olympus, Tokyo, Japan) and a digital camera ProgRes Gryphax Subra (Jenoptik, Jena, Germany). Based on cell size and coloration in micrographs, we calculated the number of cells in different zones of the developing tree ring: the cambial zone (a layer of small cells between xylem and phloem), the cell expansion zone (non-lignified cells stained blue but larger than cambium), and the zone of maturing and mature tracheids with varying degrees of lignification of the deposited secondary walls, reflected in a transition to maroon color. Tracheidograms—a series of measurements of cell size parameters along radial files—were measured to analyze the tree-ring structure. In this study, we used data on the radial diameter of cells (D) and cell wall thickness (CWT), which have the advantage of being the results of the respective differentiation phases [21]. Relationships between cell parameters and cell number per radial file were investigated using Pearson correlation coefficient (*r*), with significance estimated by two-tailed *t*-test.

The daily growth rates of trees were estimated using the process-based VS-model. Its internal calculations include several characteristics important for interpreting fluctuations in external factors and their impact on growth—soil moisture dynamics, transpiration rate, and limitation by temperature, humidity, and/or insolation (daylength) [8,22,23,24,25,26,27,47,48]. The VS-model was used in accordance with the following steps: (1) parameterization of the model using daily data from the Minusinsk weather station and the previously constructed standard local chronology of pine growth for the study area [49]; (2) assessment of the quality of modeling using statistical characteristics: the Pearson correlation coefficient and the synchronicity coefficient (GLK) between the modeled and measured chronology, as well as the root mean square error (RMSE) [47]; (3) calculation and construction of seasonal variability curves of variables that are stressful for arid conditions: soil wetness and transpiration. VS-model was calculated using free software VS-oscilloscope 1.37 (http://vs-genn.ru/downloads/, accessed on 18 December 2025), and values of its parameters that were selected during parameterization process to provide good fit of model with actual chronology are listed in Table A1.

## 5. Conclusions

An analysis of the seasonal tree-ring growth kinetics of Scots pine trees with IADF at the end of the earlywood zone was conducted and compared with the daily climate variables, soil wetness, and transpiration rates derived from the VS-model. It was shown that the intra-seasonal intervals of the observed adverse weather conditions in June–July (a cold snap, followed by drought and a heat wave) precisely correspond to the timing of production in the cambial zone for cells that record the impact of these stresses in their structure. Cells produced before or after these climatic disturbances did not change their radial size or cell wall thickness from values normal for the respective tree-ring zone. This means that the climatic signal was perceived by cells when being in the cambial zone and afterwards recorded during processes of further differentiation.

Such mechanistic understanding of the climatic impact on tree-ring formation in phenological terms should be further investigated. After outlining its limitations and interaction with other external and intrinsic factors, it can be applied in the modeling of tree growth and the improvement of dendroclimatological reconstructions based on wood structure.

## Figures and Tables

**Table 1:** Anatomical structure of tree ring developed in 2024 for seven trees, arranged in order of increasing N.

Tree #	Mean Cell Number per Radial File, N	Cell Numbers in Zones of Tracheidogram ^1^ Normalized to		Maximum Cell Wall Thickness, CWT (μm)
		Mean N	N = 15 Cells	
#2	18	12—2—4	10—2—3	3.5
#4	18	12—2—4	10—2—3	3.5
#7	19	13—2—4	11—2—2	4.0
#6	30	22—2—6	11—2—2	4.2
#3	34	22—2—10	10—1—4	5.3
#1	36	24—3—9	11—1—3	5.2
#5	52	27—6—19	9—2—4	6.3

^1^ Cell numbers separated by dashes are presented for (1) typical earlywood tracheids, (2) anomalous cells in IADF, and (3) typical tracheids of transition wood and latewood after IADF.

**Table 2:** Summary of climatic conditions and seasonal kinetics (cell numbers at various differentiation stages and temporal intervals when those stages began and ended for zones of IADF, before and after it) for the fast-growing Scots pine (tree #5) in July–August 2024. Note that the other six trees had similar patterns in the images of the forming tree ring (data not presented) at those dates, albeit with lesser cell numbers in all zones.

Calendar Dates *	Climatic Conditions	Tree-Ring Zones and Cell Differentiation Stages **		
		EW (27 Cells)	IADF (6 cells)	TW + LW (19 Cells)
≤3 July	very little rain since 10 June, severe heat wave since 22 June	start of C, E, W; end of C	start of C	–
*4 July*		*2C + 3E + 6W + 16M*	*?C*	*–*
3–15 July	heat subsided and heavy rain on 10 July	end of E	start of E, W; end of C	start of C
*16 July*		*3W + 24M*	*4E + 2W*	*?C*
17–30 July	mild heat, heavy rains on 18 July and 22 July	end of W	end of E	start of E, W
*31 July*		*27M*	*6W*	*?C + 6E + 2W*
1–15 August	mild heat and repeated small rains	–	end of W	–
*16 August*		*27M*	*1W + 5M*	*?C + 4E + 12W*
≥17 August	mild heat until 30 August and repeated small rains	–	–	end of C, E, W

* Entries in italics mark dates of direct observations of the seasonal kinetics. ** Tree-ring zones are normal earlywood cells (EW), intra-annual density fluctuation (IADF) zones, and normal transition wood and latewood (TW + LW) cells. Numbers in parentheses refer to final (at the end of season) average numbers of tracheids belonging to respective zones. Cell differentiation stages are cell production (division) in cambial zone (C); cell growth by expansion (E); secondary cell wall deposition and lignification (W); and mature tracheids (M). Cells being produced in cambial zone are marked with question mark when their number is uncertain.

## Data Availability

The original contributions presented in this study are included in the article and Supplementary Material. Used microphotographs of wood development are included as Supplementary Material. Original data on tree-ring width are available in the ITRDB (https://doi.org/10.25921/jmjk-dv97; accessed on 23 January 2026). Climatic data were obtained from the All-Russian Research Institute of Hydrometeorological Information—World Data Center (http://meteo.ru/; accessed on 25 November 2025). VS-model’s implementation named ‘VS-oscilloscope’ is available online (http://vs-genn.ru/downloads/; accessed on 18 December 2025), and the implemented values of its parameters are listed in Table A1. Further inquiries can be directed to the corresponding author.

## Supplementary Materials

The following supporting information can be downloaded at: https://www.mdpi.com/article/10.3390/plants15030348/s1, Data archive of microphotographs used in this study.

## Abbreviations

The following abbreviations are used in this manuscript:

IADF	intra-annual density fluctuation
N	number of tracheids per radial file in tree ring
D	cell radial diameter
CWT	cell wall thickness
*r*	correlation coefficient
*p*	significance level
DOY	day of year
VS-model	Vaganov-Shashkin model
GLK	synchronicity coefficient
RMSE	root mean square error
a.s.l.	above sea level
DBH	diameter at breast height (of the tree trunk)

## Appendix A

**Table A1:** Optimal parameters of the VS-model fitted for Scots pine standard tree-ring width chronology in the study area.

Parameter	Description	Value
*T_min_*	Minimum daily temperature (low threshold) for tree growth (°C)	7
*T_opt1_*	Lower end of range of optimal daily temperatures for tree growth (°C)	12
*T_opt2_*	Upper end of range of optimal daily temperatures for tree growth (°C)	24
*T_max_*	Maximum daily temperature (upper threshold) for tree growth (°C)	29
*W_min_*	Minimum soil wetness (low threshold) for tree growth (volume ratio of water to soil)	0.0775
*W_opt1_*	Lower end of range of the optimal soil wetness for tree growth (ratio)	0.35
*W_opt2_*	Upper end of range of the optimal soil wetness for tree growth (ratio)	0.425
*W_max_*	Maximum soil wetness (upper threshold) for tree growth (ratio)	0.45
*W_0_*	Initial soil wetness (ratio)	0.3
*T_beg_*	Temperature sum for initiation of growth (°C)	70
*t_beg_*	Time period for calculation of temperature sum (days)	10
*l_r_*	Depth of root system (mm)	500
*P_max_*	Maximum daily precipitation for saturated soil (mm/day)	47
*C*_1_	Fraction of precipitation penetrating soil (not caught by crown; relative unit)	0.6
*C*_2_	First coefficient for calculation of the transpiration * (mm/day)	0.155
*C*_3_	Second coefficient for calculation of the transpiration (relative unit per °C)	0.125
*Λ*	Coefficient for water drainage from soil (relative unit)	0.005

* In the VS-model, the transpiration is calculated from the daily growth rate of trees and temperature with a simplified equation, namely, *C*_2_·*Gr*·exp(*C*_3_·*T*).
