# Supplementary figures and images for: Direct Observation of the Developing Intra-Annual Density Fluctuation (IADF) for Scots Pine in Semiarid Siberian Belt Forest: External Stress Targets Cambium
Source: Plants (Basel). 2026 Jan 23;15(3):348. doi: 10.3390/plants15030348 (PMC12899285; doi:10.3390/plants15030348)

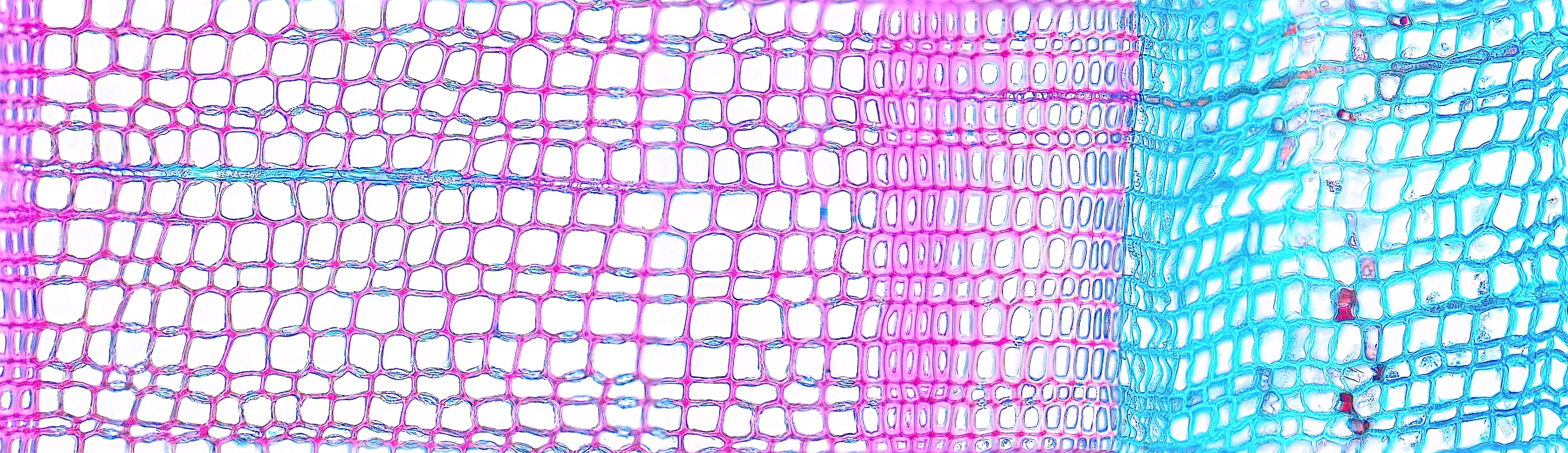

Supplement: Supplementary file 1 [file plants-15-00348-s001.zip › tree#1 10Sep.jpg]

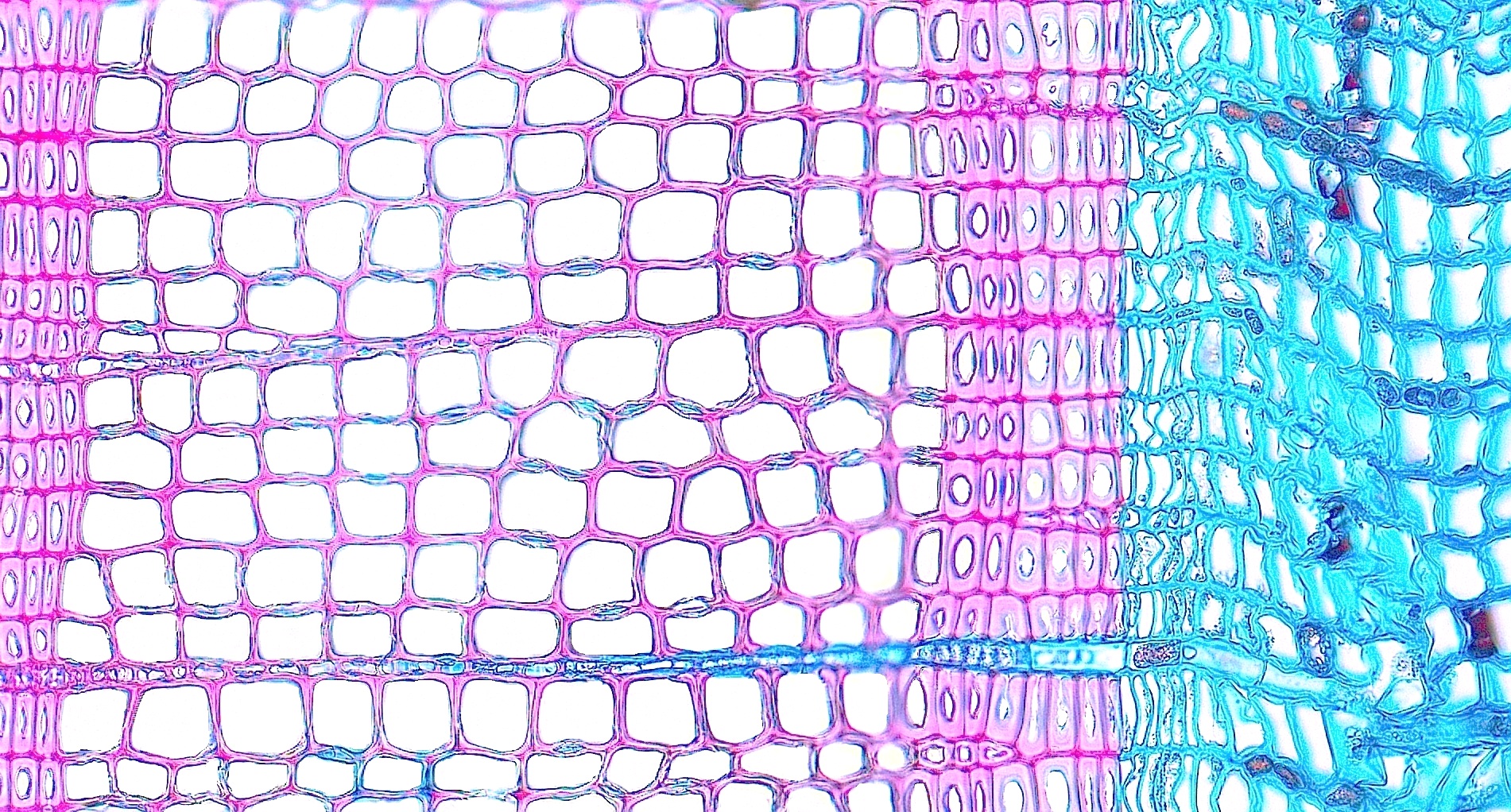

Supplement: Supplementary file 1 [file plants-15-00348-s001.zip › tree#2 10Sep.jpg]

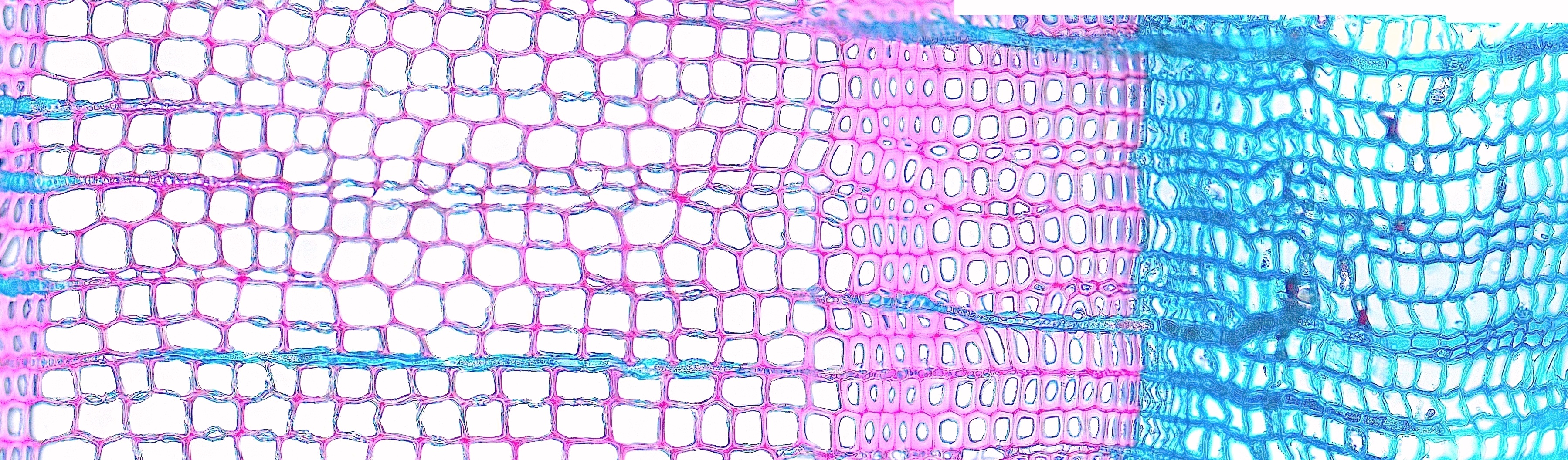

Supplement: Supplementary file 1 [file plants-15-00348-s001.zip › tree#3 10Sep.jpg]

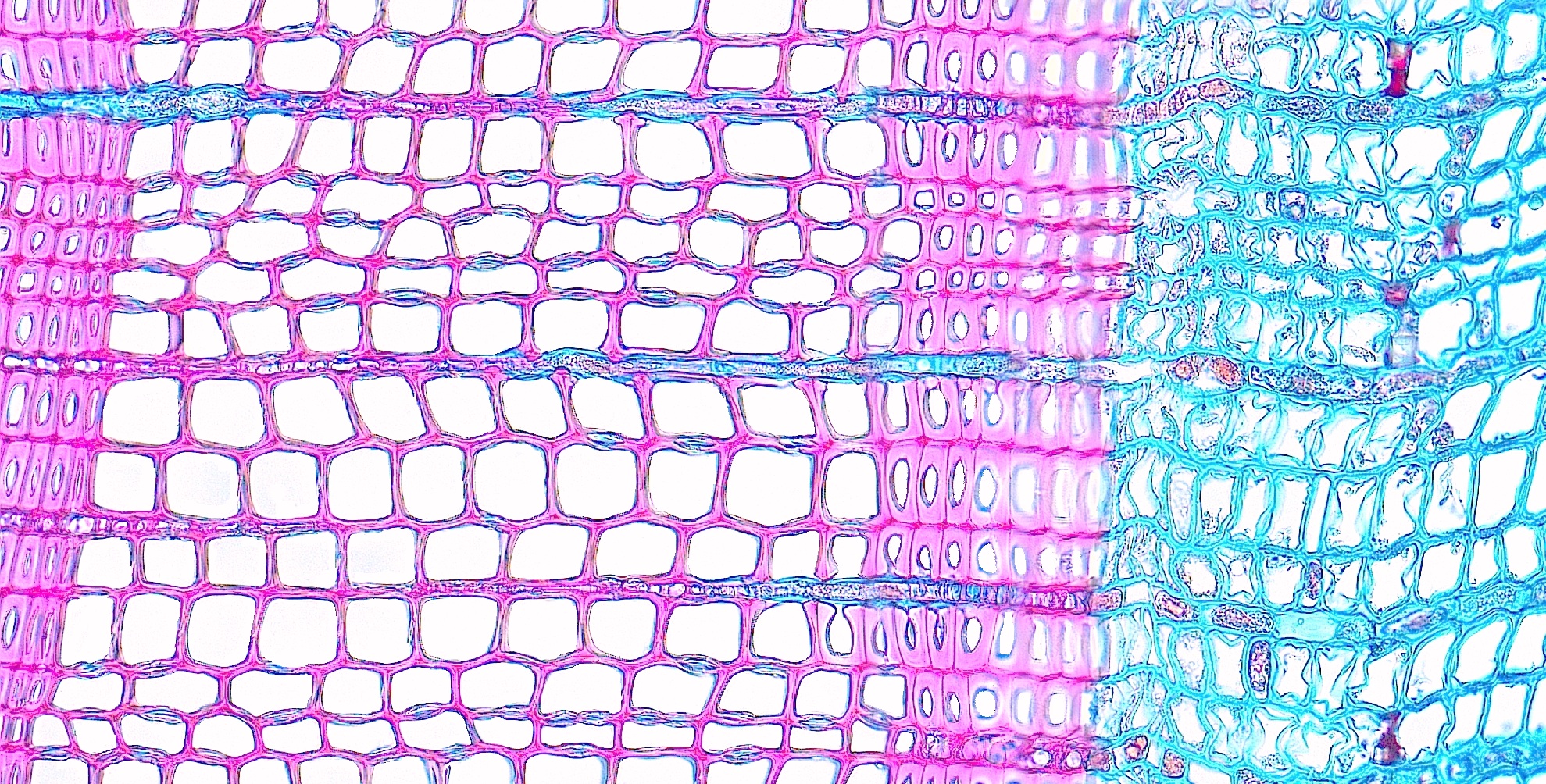

Supplement: Supplementary file 1 [file plants-15-00348-s001.zip › tree#4 10Sep.jpg]

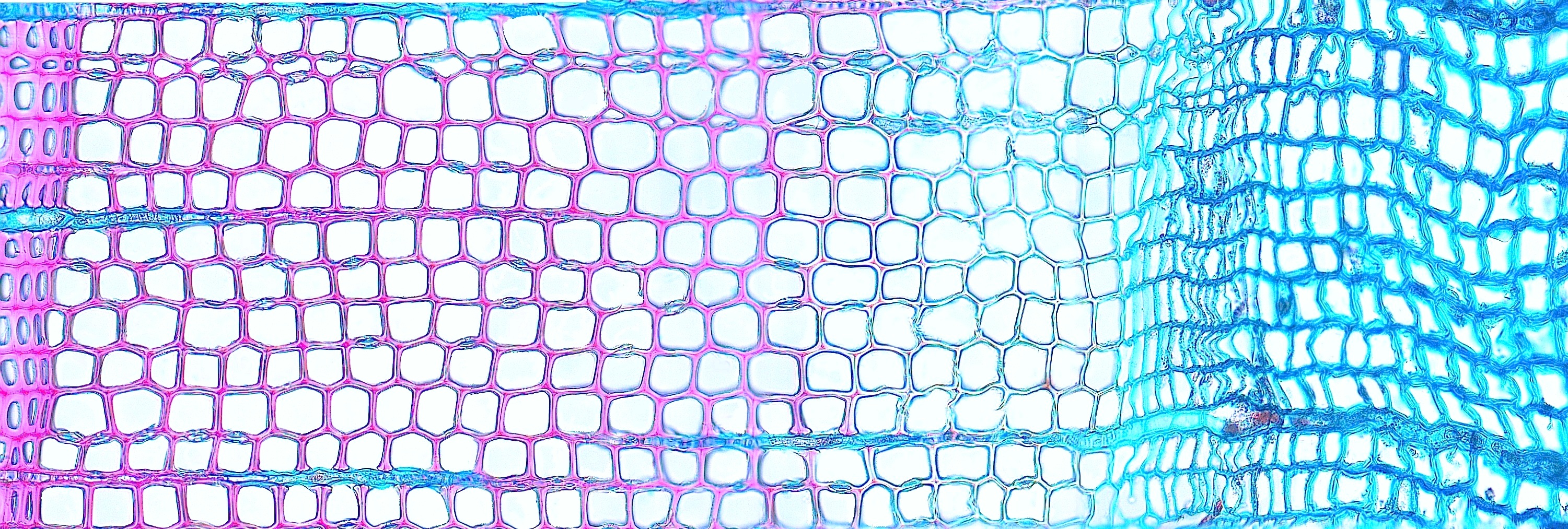

Supplement: Supplementary file 1 [file plants-15-00348-s001.zip › tree#5 04Jul.jpg]

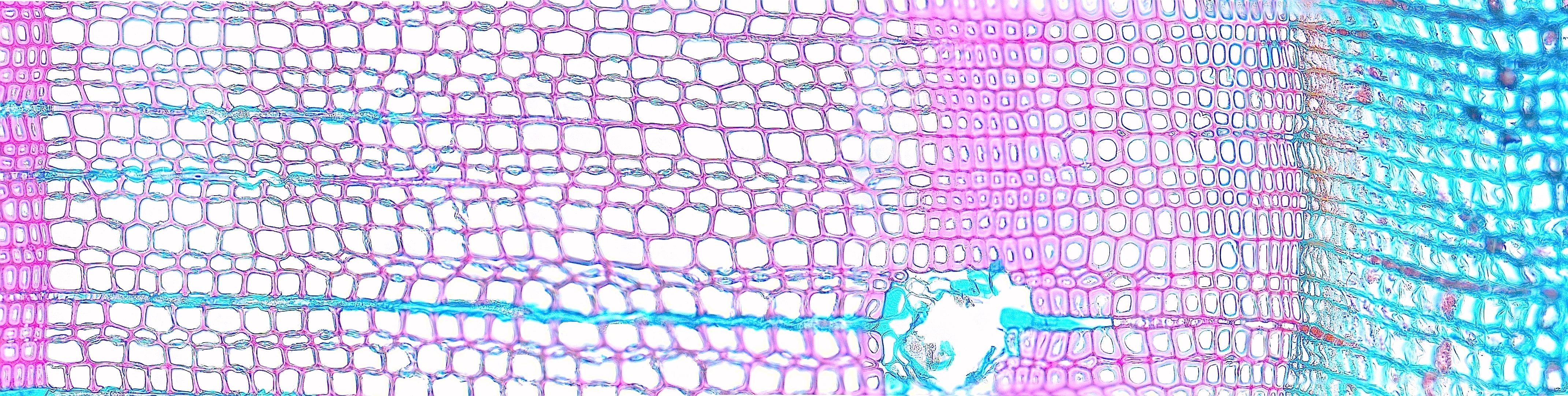

Supplement: Supplementary file 1 [file plants-15-00348-s001.zip › tree#5 10Sep.jpg]

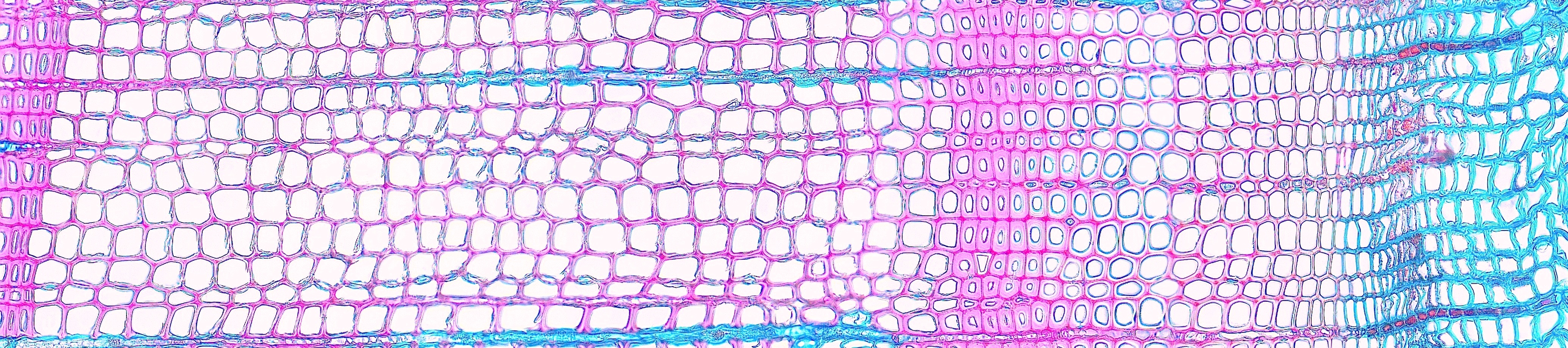

Supplement: Supplementary file 1 [file plants-15-00348-s001.zip › tree#5 16Aug.jpg]

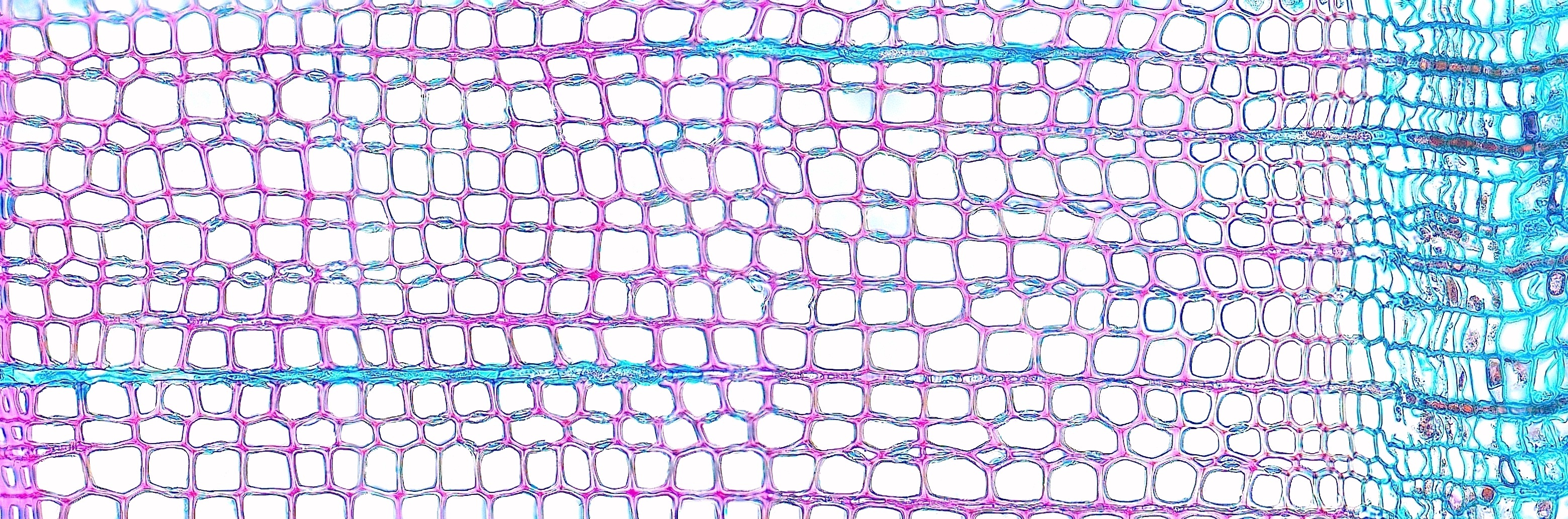

Supplement: Supplementary file 1 [file plants-15-00348-s001.zip › tree#5 16Jul.jpg]

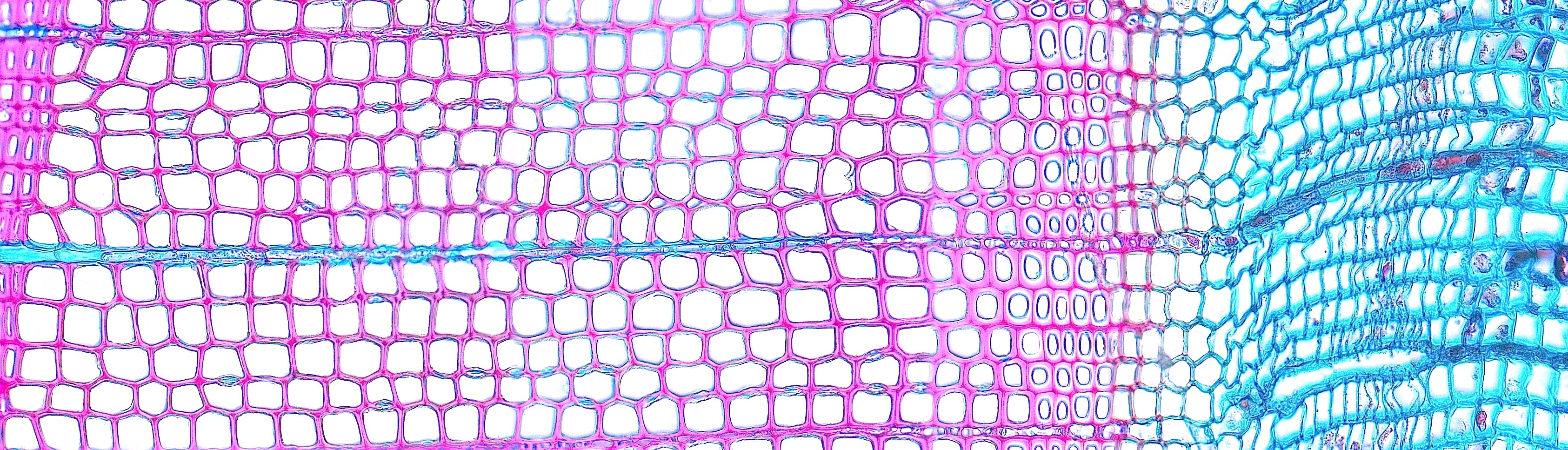

Supplement: Supplementary file 1 [file plants-15-00348-s001.zip › tree#5 31Jul.jpg]

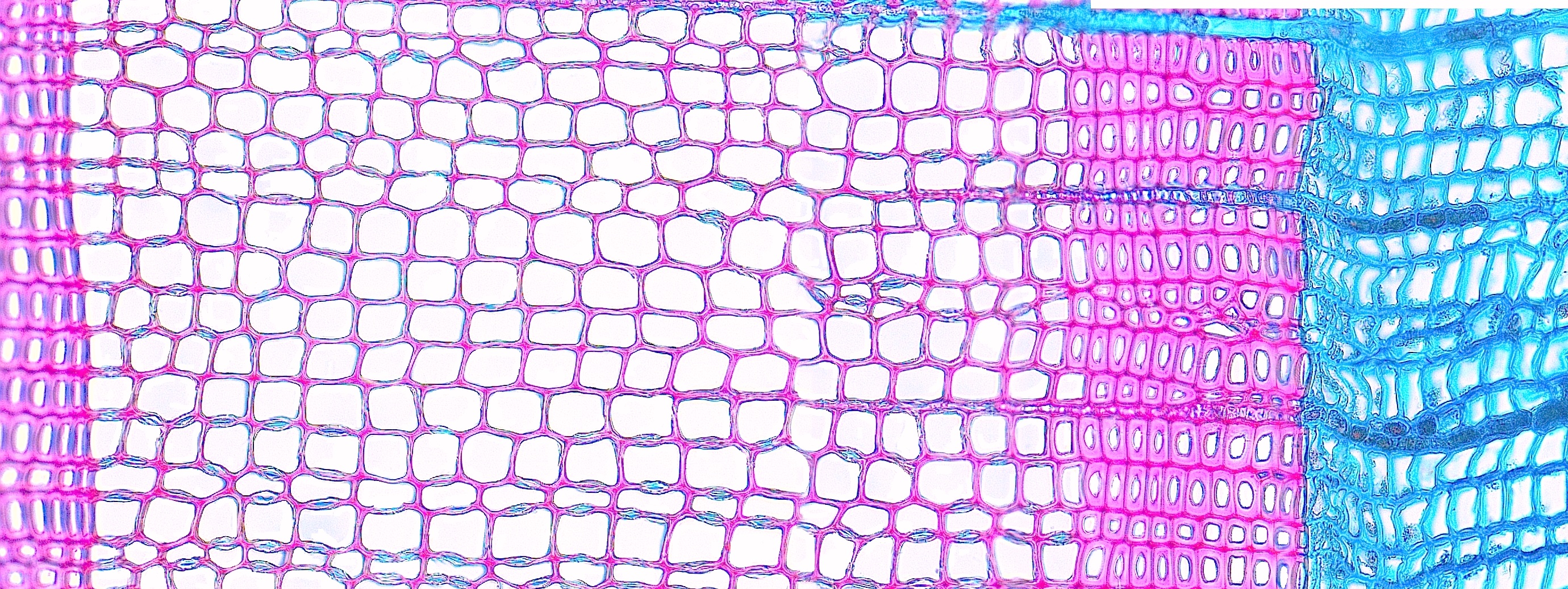

Supplement: Supplementary file 1 [file plants-15-00348-s001.zip › tree#6 10Sep.jpg]

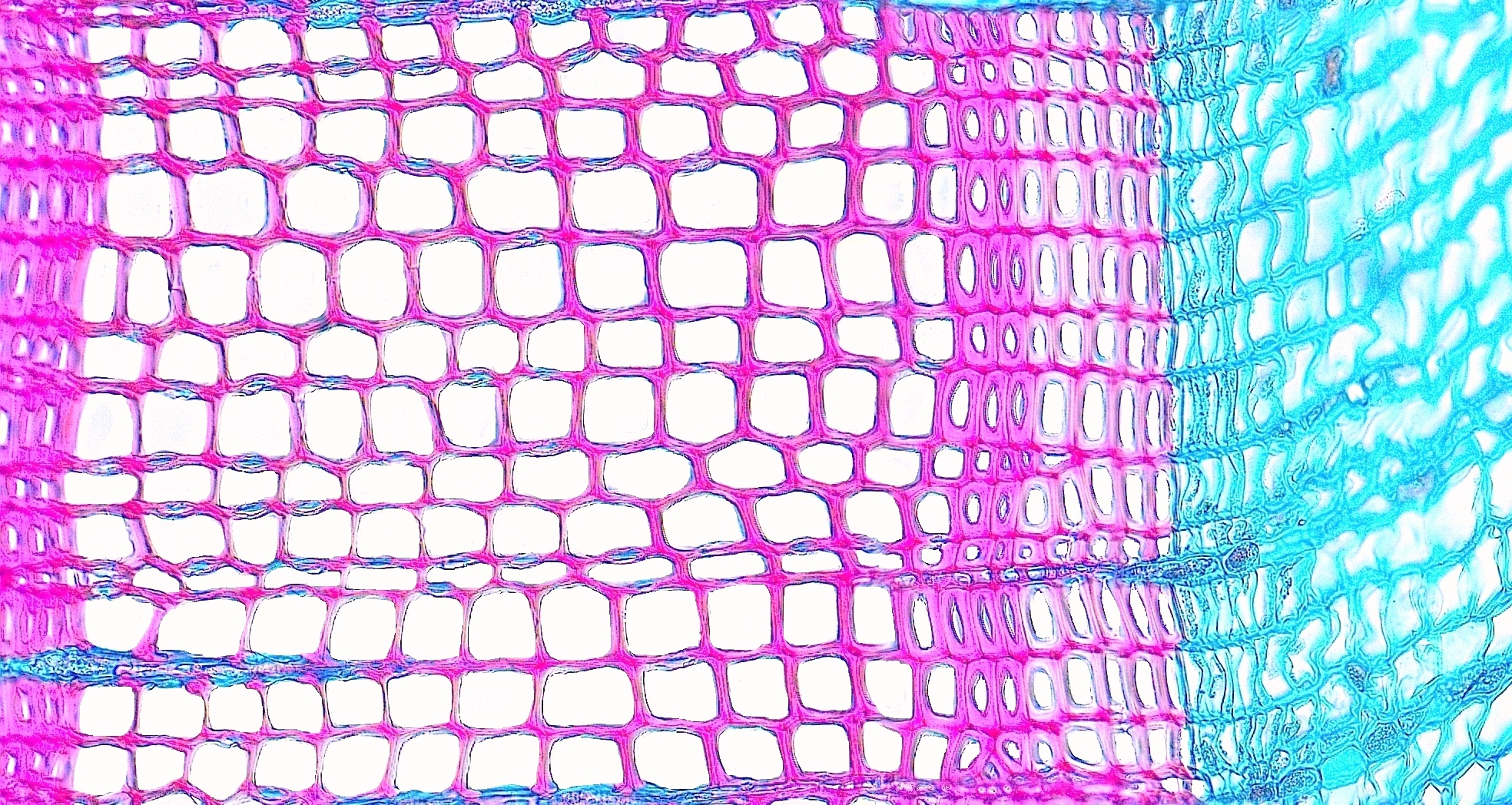

Supplement: Supplementary file 1 [file plants-15-00348-s001.zip › tree#7 10Sep.jpg]
